# Association Between Eating Habits and Perceived School Performance: A Cross-Sectional Study Among 46,455 Adolescents From 42 Countries

**DOI:** 10.3389/fnut.2022.797415

**Published:** 2022-02-03

**Authors:** José Francisco López-Gil, Arthur Eumann Mesas, Celia Álvarez-Bueno, Carlos Pascual-Morena, Alicia Saz-Lara, Iván Cavero-Redondo

**Affiliations:** ^1^Health and Social Research Center, Universidad de Castilla-La Mancha, Cuenca, Spain; ^2^Postgraduate Program in Public Health, Universidade Estadual de Londrina, Londrina, Brazil; ^3^Rehabilitation in Health Research Center (CIRES), Universidad de las Américas, Santiago, Chile

**Keywords:** nutrition, healthy eating, diet quality, adolescence, youths, academic achievement, epidemiology, international study

## Abstract

**Purpose:**

This study analyzed the association between selected self-reported eating habits and perceived school performance in adolescents by gender.

**Methods:**

A cross-sectional analysis was conducted with data from a large representative sample of adolescents from 42 different countries. Participants answered questions about their weekly frequency of fruit, vegetable, sweets, and soft drink consumption, as well as the frequency of breakfast consumption and family meals. The adolescents subjectively rated their school performance compared to that of their classmates. Logistic regression models were adjusted for region, age, body mass index (z-score), socioeconomic status, physical activity, recreational screen time, and sleep difficulties.

**Results:**

Among the 46,455 (53.5% female, mean age of 13.7 ± 1.6 years) adolescents studied, 20.6% of males and 25.5% of females reported high perceived school performance. In the results of the fully adjusted analyses, the higher the frequency of all healthy eating habits studied, the higher the perceived school performance in both males and females. Specifically, both males and females reporting a higher frequency of fruit and vegetable consumption, a lower frequency of sweets and soft drink consumption, more frequent breakfast consumption, and more frequent family meals (breakfast and dinner) were more likely to perceive their school performance as higher compared to their classmates. In addition, having breakfast regularly on weekends and the frequency of family dinner were associated with better school performance in both males and females.

**Conclusions:**

In summary, this study provide cross-sectional evidence on the association between healthy eating habits and perceived school performance. Considering that school performance is an indicator of healthy development in adolescence, our findings reinforce and extend the evidence on the importance of healthy eating at this stage of life.

## Introduction

Adolescence is characterized by rapid physical, cognitive, and psychosocial growth, which affects how they deal with their own feelings, thoughts, decision-making processes, and interactions with the world around them (1). The biological and psychosocial changes that occur at this stage of life produce one of the greatest needs for nutrients throughout the life cycle (2). However, because of the transition to greater independence from their parents regarding food choices, adolescents are more vulnerable in terms of nutrition than younger children (3). Similarly, during adolescence, peer, and media influences exert a considerably greater impact on food choices, frequently in favor of foods with less healthy nutritional content (2).

Furthermore, adolescence is a crucial phase for brain maturation, as myelination, synaptic pruning and several neural connections develop, particularly in the prefrontal cortex (4, 5). This brain maturation is accompanied by the emergence of increasingly sophisticated cognitive abilities, which, in turn, are bidirectionally associated with school performance (6). In addition, nutrition is one of the most modifiable aspects of lifestyle that can influence brain maturation and, consequently, cognition and school performance (7). In this regard, a previous longitudinal study conducted over a 3-year period showed that lifestyle habits (e.g., eating breakfast) were related to cognitive control and school performance in adolescents (8). Likewise, a systematic review showed moderate relationships of school performance with better overall diet quality, as well as with healthier eating habits, such as regular breakfast consumption and lower consumption of energy-dense foods and foods with poor nutritional value (9).

Given the above, a deeper understanding of whether eating habits are related to school performance could be crucial for parents/legal guardians, public health researchers, and policy-makers (10). However, research on the association between dietary patterns and school performance is still developing and has certain limitations (9). For instance, some studies analyzed the relationship between school performance and eating habits among adolescents, but only in a single country [e.g., Canada (11) or Australia (12)]. Furthermore, although most studies examining the association between school performance and eating habits have focused on breakfast consumption (9, 13), studies assessing the relationship with other eating habits (e.g., family meals) are scarce (11). Thus, the present study examined the association between multiple eating habits (including fruit, vegetable, sweets, and soft drink consumption, as well as having breakfast and family meals) and school performance among a large, representative sample of adolescents from 42 different countries.

## Methods

### Study Design and Population Sample

This is a cross-sectional study using data from 42 countries (Albania, Armenia, Austria, Belgium (Flemish), Belgium (French), Bulgaria, Canada, Switzerland, Czech Republic, Germany, Denmark, Estonia, England, Spain, Finland, France, Greenland, Greece, Croatia, Hungary, Ireland, Israel, Iceland, Italy, Lithuania, Luxembourg, Latvia, Republic of Moldova, North Macedonia, Malta, Netherlands, Norway, Poland, Portugal, Romania, Russian Federation, Scotland, Sweden, Slovenia, Slovakia, Ukraine, Wales) from the 2013/2014 wave of the ongoing international Health Behavior in School-Aged Children (HBSC) study, which includes nationally representative samples of adolescents aged 10–17 years (14). Adolescents were randomly selected from their schools and anonymously completed a standardized questionnaire, which had been translated into their local language. Students were free to leave any question unanswered. Institutional ethical authorization was received from each participating country. Moreover, both the schools and the adolescents and their parents or legal guardians received and signed written informed consent forms.

The present analysis includes data from all the countries that provided information on diet habits, school performance, and the covariates considered. The total number of participants was 214,175, of whom 167,720 (78.3%) were excluded because of missing data on some of the study variables, resulting in a final sample of 46,455 (53.5% females) adolescents from 42 different countries.

### Procedures

#### Perceived School Performance

Perceived school performance was evaluated using the following question: “In your opinion, what does your class teacher(s) think about your classroom performance compared to your classmates?” For this question, the response options were very good, good, average, and below average. Subsequently, the different options were collapsed into high perceived school performance (very good) and not high perceived school performance (good, average, and below average).

#### Eating Habits

Four different eating habits were measured using variations of the following basic question: “How many times a week do you consume fruits?” (Response options were never, less than once a week, 2–4 times a week, 5–6 times a week, once daily, more than once daily). This question format and the same response options were then used to assess the consumption of vegetables, sweets, and soft drinks. Additionally, breakfast habits were evaluated by the following question: “How often do you usually have breakfast (more than a glass of milk or fruit juice)?” (Response options for weekdays were 1—never to 5—every weekday; and response options for weekends were 1—never to 3—both days). Family meals habits were assessed by the following questions: “How often do you eat breakfast together with your mother or father?” and “How often do you eat dinner together with your family?” Possible responses ranged from never to 7 days for each of the questions.

#### Covariates

Region, gender, age, weight, and height were self-reported by the adolescents. Height and weight values were used to determine the body mass index (kg/m^2^). The body mass index z-score was determined following the specific International Obesity Task Force (IOTF) criteria (15) and, therefore, the prevalence of excess weight (≥1 SD) was determined. The Family Affluence Scale (FAS) (16) was used to assess socioeconomic status. The FAS includes questions on material goods (e.g., computers) and vacations to estimate socioeconomic status. Physical activity was measured with the following question: “Over a typical or usual week, on how many days are you physically active for a total of at least 60 min per day?” (Responses varied from 0 to 7 days per week) (17). Recreational screen time was evaluated by the following three questions: (1) “How many hours a day, in your free time, do you usually spend using electronic devices such computers, tablets (like an iPad) or smartphones for purposes such as homework, e-mailing, tweeting, using Facebook, chatting, surfing the internet?,” (2) “How many hours a day, in your free time, do you usually spend playing games on a computer, game console, tablet (like an iPad), smartphone, or other electronic device (not including moving or fitness games)?,” and (3) “How many hours a day, in your free time, do you usually spend watching TV, videos (including YouTube or similar services), DVDs, and other entertainment on a screen?” All questions included nine answer options (responses ranged from 0 to more than 7 h per day). Participants answered these questions separately for weekdays and weekends. Similarly, adolescents were asked to report the frequency of sleep difficulties using a five-point scale ranging from rarely or never to almost every day. The selection of these covariates was based on scientific evidence of their potential confounding effect on the association between school performance and eating habits (9, 18–21).

### Statistical Analysis

Data for categorical and continuous variables were expressed as numbers and percentages and as the mean and standard deviation, respectively. Odds ratios were interpreted as the likelihood of achieving “high performance” (“very good”) or “not high performance” (“good,” “average,” and “below average”) according to the different eating habits. For this purpose, multilevel mixed effects logistic regression analyses were performed including different eating habits and covariates described above in addition to country-specific random effects. Data analyses were conducted using the software Statistical Package for Social Sciences (SPSS) (Version 25.0). A *p*-value of 0.05 was used to establish statistical significance. All analyses were adjusted by region, age, body mass index (z-score), socioeconomic status, physical activity, recreational screen time, and sleep difficulties.

## Results

Table 1 shows the characteristics of the sample analyzed for males and females. The mean age was 13.7 years (SD = 1.6) for both males and females. The prevalence of excess weight (overweight and obesity) was higher in males (29.5%) than in females (19.2%) (*p* < 0.001). The proportion of adolescents reporting a high (“very good”) perceived school performance was higher in females (25.5%) than in males (20.6%) (*p* < 0.001).

**Table 1:** Characteristics of the study participants (*N* = 46,455).

**Variables**	**Males (*n* = 21,591; 46.5%)**	**Females (*n* = 24,864; 53.5%)**	***p***
	**n/M (%/SD)**	**n/M (%/SD)**	
Age (years)	13.7 (1.6)	13.7 (1.6)	0.210
Weight (kg)	54.4 (15.0)	50.0 (11.4)	<0.001
Height (cm)	163.9 (13.5)	159.5 (9.5)	<0.001
BMI (z-score)^a^	0.40 (1.15)	0.10 (1.09)	<0.001
Excess weight^a^	6,376 (29.5)	4,781 (19.2)	<0.001
FAS-III (score)	5.9 (2.0)	5.8 (2.0)	<0.001
High SES	556 (2.6)	483 (1.9)	<0.001
Medium SES	8,244 (38.2)	9,834 (35.9)	
Low SES	12,792 (59.2)	15,447 (62.1)	
**Physical activity**			
≥60 min MVPA (days)	4.4 (2.0)	3.7 (2.0)	<0.001
**Recreational screen time**			
TV/DVD/Video on weekdays (>2 h)	12,935 (59.9)	14,264 (57.4)	<0.001
TV/DVD/Video on weekends (>2 h)	16,398 (75.9)	18,649 (75.0)	<0.001
Computer games on weekdays (>2 h)	10,398 (48.2)	6,650 (26.7)	<0.001
Computer games on weekends (>2 h)	14,379 (66.6)	9,764 (39.3)	<0.001
Computer use on weekdays (>2 h)	9,569 (44.3)	11,860 (47.7)	<0.001
Computer use on weekends (>2 h)	11,766 (54.5)	14,409 (58.0)	<0.001
**Sleep difficulties**			
About every day	1,620 (7.5)	2,760 (11.1)	<0.001
**Perceived academic performance**			
Very good	4,440 (20.6)	6,332 (25.5)	<0.001
Good	9181 (42.5)	10,395 (41.8)	
Average	6,666 (30.9)	7,123 (28.6)	
Below average	1,181 (5.5)	918 (3.7)	

*BMI, body mass index; MVPA, moderate-to-vigorous physical activity; SES, socioeconomic status*.

a *According to the international obesity task force criteria (15)*.

**Figure 1:**
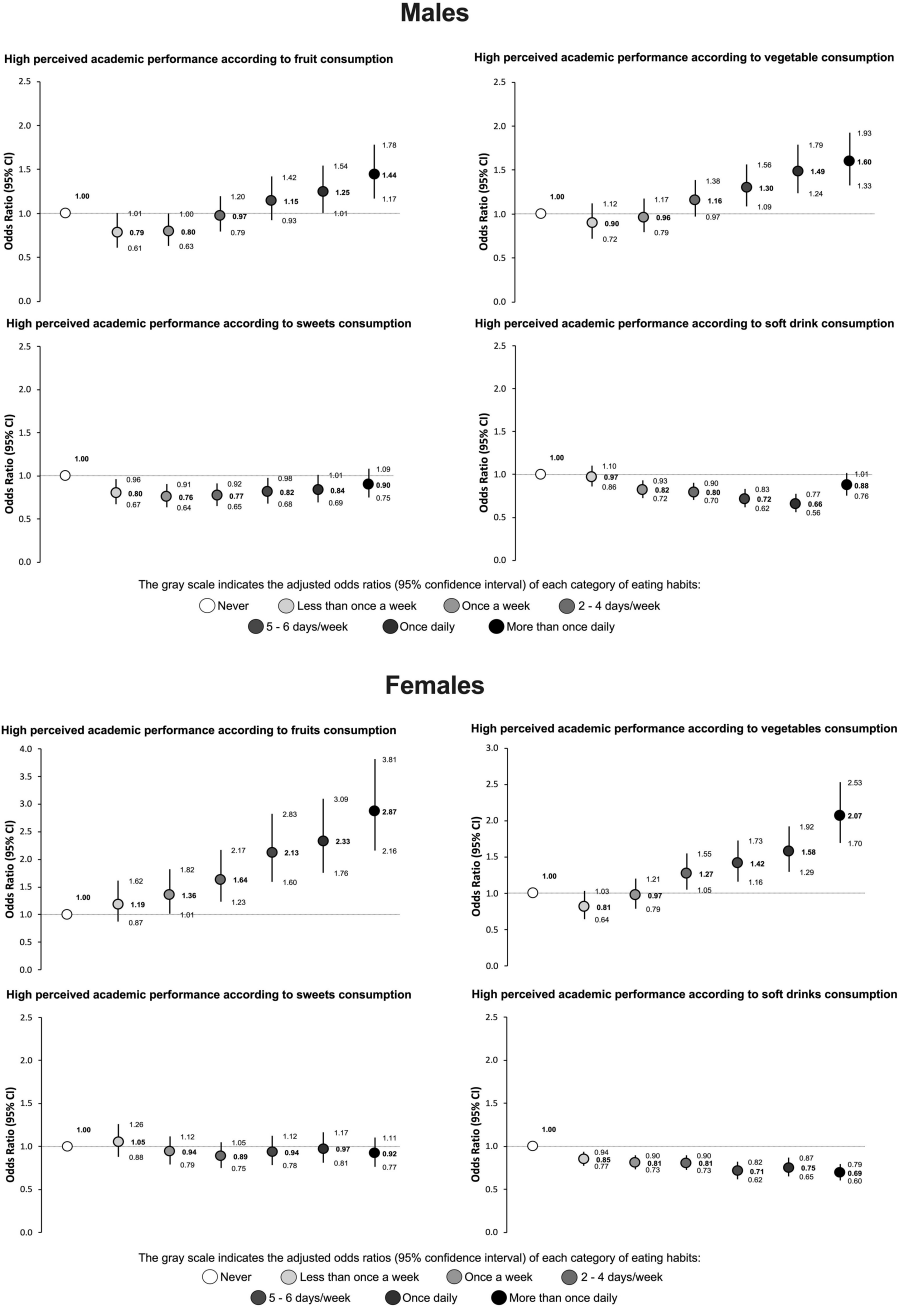
Association between high perceived academic performance and fruit, vegetable, sweets, and soft drink consumption among adolescents. Odds ratios generated using logistic regression models adjusted by region, age, body mass index (z-score), socioeconomic status, physical activity, recreational screen time, and sleep difficulties.

Figure 1 shows the fully adjusted association between high perceived school performance and the consumption of fruits, vegetables, sweets, and soft drinks. In both males and females, the highest likelihood of reporting high perceived school performance was observed among those who consumed fruits (males: OR = 1.44; 95% CI: 1.17–1.78; females: OR = 2.87; 95% CI: 2.16–3.81) and vegetables (males: OR = 1.60; 95% CI: 1.33–1.93; females: OR = 2.07; 95% CI: 1.70–2.53) more than once a day. Conversely, in general, the likelihood of reporting high performance was lower in both males and females as the frequency of sweets consumption increased. Similarly, in both males and females, it was observed that the higher the frequency of soft drink consumption, the lower the probability of reporting high performance.

**Figure 2:**
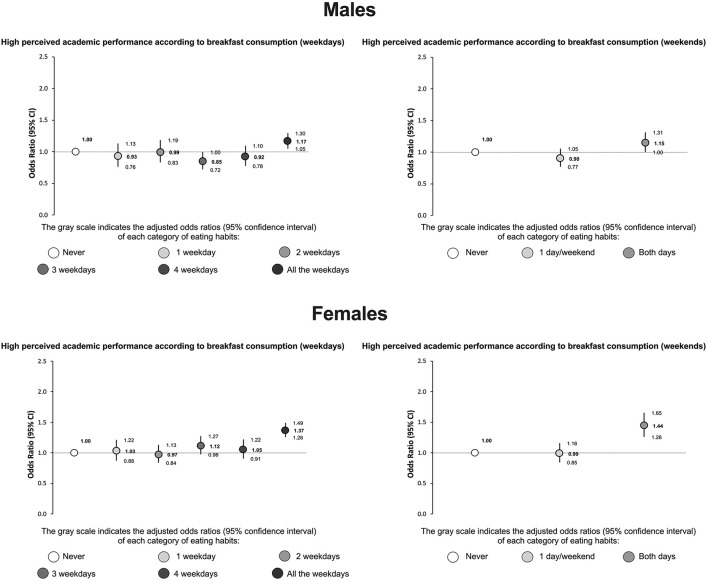
Association between high perceived academic performance and breakfast consumption (on both weekdays and weekends) among adolescents. Odds ratios generated using logistic regression models adjusted by region, age, body mass index (z-score), socioeconomic status, physical activity, recreational screen time, and sleep difficulties.

As shown in Figure 2, the dietary habit of eating breakfast daily on weekdays was associated with better school performance in both males (OR = 1.17; 95% CI: 1.05–1.30), and females (OR = 1.37; 95% CI: 1.26–1.49). Having breakfast regularly on weekends was associated with better school performance in both males (OR = 1.15; 95% CI: 1.00–1.31) and females (OR = 1.44; 95% CI: 1.26–1.65).

**Figure 3:**
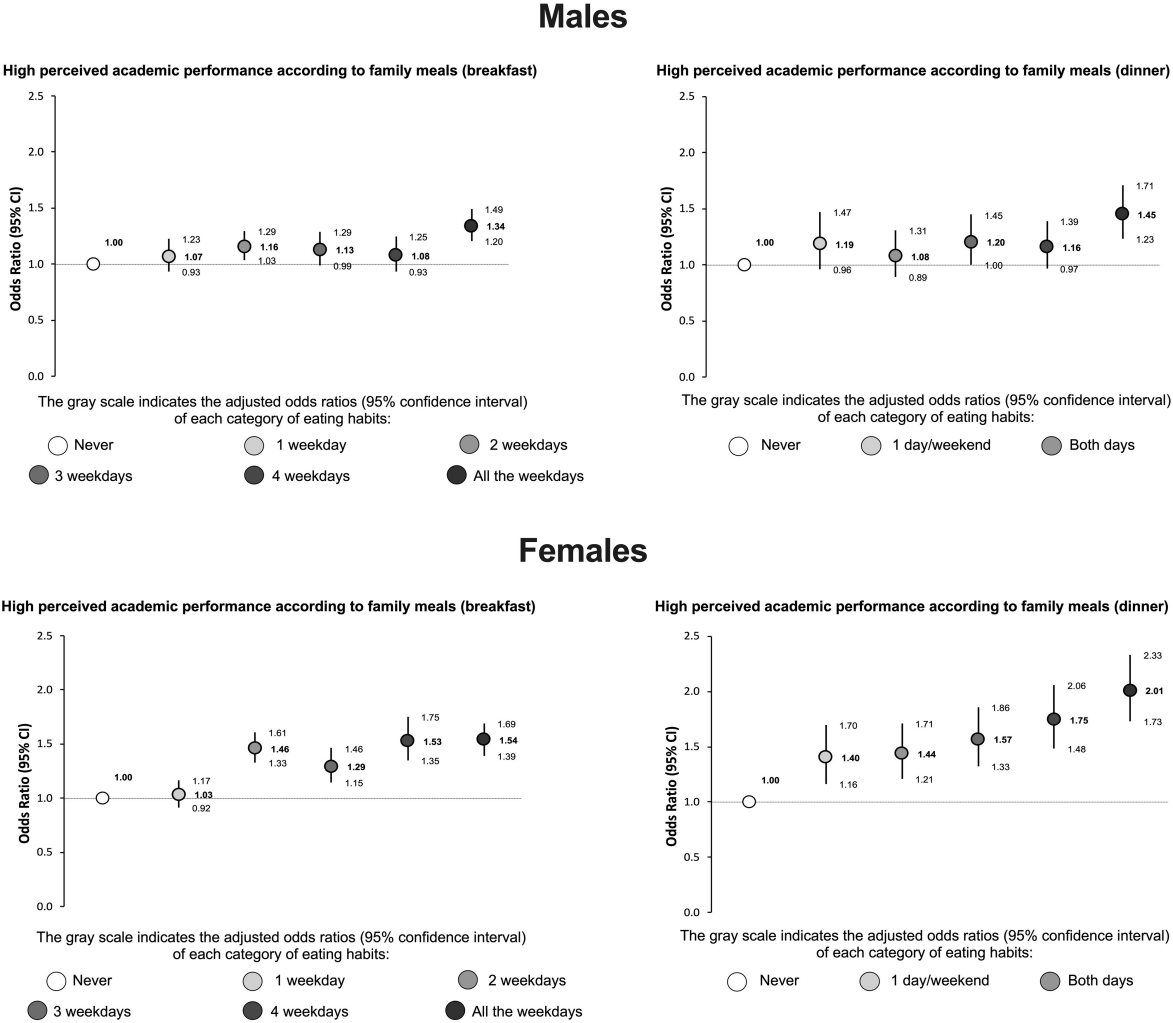
Association between high perceived academic performance and frequency of family meals (breakfast and dinner) among adolescents. Odds ratios generated using logistic regression models adjusted by region, age, body mass index (z-score), socioeconomic status, physical activity, recreational screen time, and sleep difficulties.

The relationship between high perceived school performance and the frequency of family meals (breakfast and dinner) is shown in Figure 3. In general, adolescents of both males and females who had breakfast with their families more frequently were more likely to report high school performance (males: OR = 1.34; 95% CI: 1.20–1.49; females: OR = 1.54; 95% CI: 1.39–1.69). Furthermore, although the habit of eating dinner with the family daily was associated with high school performance in both males (OR = 1.45; 95% CI: 1.23–1.71) and females (OR = 2.01; 95% CI: 1.73–2.33), the pattern of association between the frequency of family dinner and school performance was clearer in females than in males.

## Discussion

### Summary of the Main Results

The main finding of the present study was that in adolescents from several countries, all the healthy eating habits analyzed were associated with high perceived school performance. More specifically, in both males and females, it was found that the higher the frequency of healthier eating habits, the higher the perceived school performance reported. These results were consistent overall for the reported consumption of fruits, vegetables, sweets, and soft drinks and for having breakfast and family meals (breakfast and dinner).

### Fruit and Vegetable Consumption and High Perceived School Performance

Our results indicate that a more frequent consumption of fruits and vegetables was related to high perceived school performance in both males and females. These findings are in agreement with the conclusions from the systematic review conducted by Burrows et al. (9). Similarly, Tapia-Serrano et al. (22) found that adherence to the Mediterranean diet, which is characterized by frequent intake of a large number of healthy foods [e.g., vegetables and fruits (23)], was cross-sectionally associated with higher school performance in Spanish adolescents regardless of their nutritional status. Among the possible mechanisms underlying the relationship between a higher consumption of fruits and vegetables and school performance is the high density of vitamin C, which cooperates with vitamin E (also known as tocopherol) as an antioxidant (24). In this regard, Alghadir et al. (25) found a positive correlation between two subtypes of tocopherol (α- and γ-tocopherol) and executive function and school performance in adolescents. These same authors pointed out that adolescents with learning difficulties could benefit from a well-balanced diet that provides adequate levels of vitamin E and other antioxidants. Additionally, increased consumption of fruits and vegetables may also increase the intake of some micronutrients, such as folate or iron, which have been associated with improved school performance (26, 27).

### Sweets and Soft Drink Consumption and High Perceived School Performance

Furthermore, considering that sweets and soft drinks are known for their low nutritional value and high caloric value, a higher frequency of consumption of these unhealthy foods was associated with lower rates of self-reported high academic achievement. Supporting this finding, one study of Norwegian adolescents by Øverby et al. (28) showed that higher consumption of unhealthy foods was related to self-reported learning difficulties in math. Similarly, Nyaradi et al. (12) reported that the “Western” dietary pattern, which includes a higher consumption of soft drinks and ultra-processed food (among others) than other dietary patterns, was related to significantly lower school performance in Australian adolescents. One of the possible reasons for this finding is that sweets and soft drinks contain high amounts of saturated fats and free sugars, which have been associated with impaired functioning of the hippocampus, a brain structure involved in learning and memory (29). This fact is especially relevant during adolescence, since the hippocampus expands its volume at this stage of development (30).

### Breakfast Consumption and High Perceived School Performance

In addition, our results showed that a higher frequency of breakfast consumption was related to higher perceived school performance, which is consistent with the scientific literature among adolescents (9, 13). Breakfast may improve neuronal activity (31) and cognitive control (e.g., working memory and attentional capacities) (32), which in turn can influence school performance. In line with this association, Adolphus et al. (33) showed in a systematic review of studies with children and adolescents that, breakfast intake positively influences cognitive function compared to skipping breakfast. Likewise, Masoomi et al. (34) and Kawabata et al. (35) demonstrated that breakfast consumption had a positive influence on students' cognitive functions and school performance. Furthermore, Peña-Jorquera et al. (36) indicated that adolescents who eat breakfast before cognitively demanding activities and who regularly include a high-quality breakfast have higher cognitive performance than their counterparts who do not. A possible reason for these findings could lie in the association between skipping breakfast and an increased likelihood of depression, anxiety, psychological distress, and stress in adolescence (37), which can negatively affect school performance. Finally, because regular meal consumption has been associated with higher diet quality (9, 34), not skipping breakfast may help adolescents achieve a high-quality diet (38), which could then explain their high perceived school performance.

### Regular Family Meal Consumption and High Perceived School Performance

Regarding regular consumption of family meals, we found an association between a higher frequency of family meals (breakfast and dinner) and higher perceived school performance. Although the consumption of family meals has been shown to be beneficial for the health and psychosocial well-being of adolescents (39) (which could be key factors in improving school performance), we found only a single study that examined this eating behavior in terms of school performance among Canadian adolescents (11). These same authors showed that healthy eating habits (e.g., family meals) were positively associated with higher academic achievement, which is consistent with our study results. In this sense, Eisenberg et al. (39) suggested that family meals could provide a formal/informal time during which parents could connect with their children's emotional well-being, which could at least partially explain the associations found in our analysis. Another rationale could be related to the association between family meals and higher quality of diet. Family meals are associated with a higher consumption of healthy foods, such as fruits and vegetables (40, 41), and with a better overall diet as assessed with the Healthy Eating Index (40), which may be related to high perceived school performance. Furthermore, another possible explanation is that family meals may be an overall proxy for parental availability and involvement in their child's life, with could provide additional support for academic achievement (42).

### Methodological Considerations

The main strength of the study is the large and representative sample of adolescents analyzed from 42 countries, which confers substantial external validity to our findings. Although the statistical significance of small effect sizes can be demonstrated in studies analyzing large datasets, the results from this study provide evidence of the association between eating habits and perceived school performance. Nevertheless, because of the cross-sectional study design, we cannot establish a cause-effect relationship. Thus, future prospective observational and experimental studies based on objective measures are required to examine whether an increased frequency of healthy dietary eating habits leads to improved school performance in adolescents. Although the measures used have been previously validated, the questions asked were brief—intended to reduce the burden of questions on participants—and did not provide in-depth data on the variables analyzed. A more detailed measurement would provide additional information for each item, as well as information about other healthy foods such as nuts, seeds, pulses, or diaries. Also, the assessment of cognitive ability would be interesting, since it has been related to healthier lifestyle habits (e.g., eating habits) (43). Likewise, weight and height were parent-reported which could introduce error and bias to the results obtained (44). Moreover, information on dietary patterns and school performance may result in some differential bias because of information and recall bias, social desirability bias or overestimation by adolescents. Furthermore, the question on perceived school performance asked the participants to speculate about what their teacher thought about them, relative to their classmates, which may lead to different interpretations. In addition, although we controlled our analysis for the effect of important confounders, including sociodemographic, anthropometric, and lifestyle variables related to movement, such as physical activity, screen use, and sleep, residual confounding is still possible. Lastly, for the present study we used data collected in 2013/2014. However, the most recent information from the HBSC study is not publicly available (at this time).

## Conclusions

In conclusion, this study provides cross-sectional evidence that selected healthy eating habits, such as a higher frequency of fruit and vegetable consumption, a lower frequency of sweets and soft drink consumption, more frequent breakfast consumption, and more frequent family meals (breakfast and dinner), are associated with high perceived school performance among adolescents, regardless of the main confounding factors. Considering the importance of school performance for the cognitive, emotional and physical development of adolescent students, it is necessary to continue to raise awareness and develop intervention programs that consider the promotion of healthy eating habits.

## Data Availability Statement

The datasets presented in this study can be found in online repositories. The names of the repository/repositories and accession number(s) can be found below: https://www.uib.no/en/hbscdata.

## Ethics Statement

Ethical review and approval was not required for the current study in accordance with the local legislation and institutional requirements. Written informed consent from the participants was not required for the current study in accordance with the national legislation and the institutional requirements. For the datasets on which this study is based, each participant country is responsible for researching under their ethical guidelines, consequently, consent to carry out the research was given by school administrators in each country. Moreover, both the schools and the adolescents and their parents or legal guardians received and signed written informed consent forms.

## Author Contributions

JL-G designed the study, contributed to the interpretation of the data, and to the analysis and writing of the draft. AM, CÁ-B, CP-M, AS-L, and IC-R contributed to the revision of the manuscript. All authors approved the final version of the manuscript.

## Conflict of Interest

The authors declare that the research was conducted in the absence of any commercial or financial relationships that could be construed as a potential conflict of interest.

## Publisher's Note

All claims expressed in this article are solely those of the authors and do not necessarily represent those of their affiliated organizations, or those of the publisher, the editors and the reviewers. Any product that may be evaluated in this article, or claim that may be made by its manufacturer, is not guaranteed or endorsed by the publisher.
